# PIPE4: Fast PPI Predictor for Comprehensive Inter- and Cross-Species Interactomes

**DOI:** 10.1038/s41598-019-56895-w

**Published:** 2020-01-29

**Authors:** Kevin Dick, Bahram Samanfar, Bradley Barnes, Elroy R. Cober, Benjamin Mimee, Le Hoa Tan, Stephen J. Molnar, Kyle K. Biggar, Ashkan Golshani, Frank Dehne, James R. Green

**Affiliations:** 1 Department of Systems and Computer Engineering, Carleton University, Ottawa, Ontario K1S 5B6 Canada; 2 Agriculture and Agri-Food Canada, Ottawa Research and Development Centre, Ottawa, Ontario K1A 0C6 Canada; 3 Department of Biology, Carleton University, Ottawa, K1S 5B6 Ontario Canada; 4 Agriculture and Agri-Food Canada, Saint-Jean-sur-Richelieu Research and Development Centre, Saint-Jean-sur-Richelieu, J3B 3E6 Quebec Canada; 5 Ottawa Institute of Systems Biology, Carleton University, 1125 Colonel By Drive, Ottawa, K1S 5B6 Canada; 6 School of Computer Science, Carleton University, Ottawa, Ontario K1S 5B6 Canada

**Keywords:** Computational biology and bioinformatics, Protein function predictions

## Abstract

The need for larger-scale and increasingly complex protein-protein interaction (PPI) prediction tasks demands that state-of-the-art predictors be highly efficient and adapted to inter- and cross-species predictions. Furthermore, the ability to generate comprehensive interactomes has enabled the appraisal of each PPI in the context of all predictions leading to further improvements in classification performance in the face of extreme class imbalance using the Reciprocal Perspective (RP) framework. We here describe the PIPE4 algorithm. Adaptation of the PIPE3/MP-PIPE sequence preprocessing step led to upwards of 50x speedup and the new Similarity Weighted Score appropriately normalizes for window frequency when applied to any inter- and cross-species prediction schemas. Comprehensive interactomes for three prediction schemas are generated: (1) cross-species predictions, where *Arabidopsis thaliana* is used as a proxy to predict the comprehensive *Glycine max* interactome, (2) inter-species predictions between *Homo sapiens-*HIV1, and (3) a combined schema involving both cross- and inter-species predictions, where both *Arabidopsis thaliana* and *Caenorhabditis elegans* are used as proxy species to predict the interactome between *Glycine max* (the soybean legume) and *Heterodera glycines* (the soybean cyst nematode). Comparing PIPE4 with the state-of-the-art resulted in improved performance, indicative that it should be the method of choice for complex PPI prediction schemas.

## Introduction

The elucidation of protein-protein interaction (PPI) networks is central to molecular biology research. Necessary to producing mechanistic models of cellular processes, PPI networks additionally contribute to challenges such as the prediction of gene function^1–3^, identification of disease genes^4^, and pharmaceutical discovery^5,6^. Computational PPI prediction techniques have been developed to supplement and guide wet-laboratory experimental work. The last decade has seen increased computational demand in both scale and complexity of PPI predictors. Predicting comprehensive interactomes (the set of all possible pairwise PPIs in or between proteomes) has only recently become possible with the advent of high-performance computing infrastructure and algorithmic optimizations. While methodologically diverse in their implementation, PPI prediction tools generally exploit information from the set of known PPIs (previously confirmed using classical wet-laboratory techniques) to determine whether any two query proteins will physically interact. The utility and scalability of any one method is subject to the information it leverages.

Structure-based methods, at one extreme, require the three-dimensional (3D) characterization of each protein and therefore suffer from low coverage of the proteome. While useful to determining highly specific PPI networks, many methods require template-based modelling which tend to be computationally taxing^7–9^. Furthermore, even with complete 3D structural information of each protein in an organism’s proteome, the computational time complexity to elucidate a single putative PPI make these methods prohibitive beyond modestly sized networks^10^. At the other extreme, sequence-based predictors rely solely upon primary sequence data making them amenable to the investigation of proteome-wide networks. Furthermore, these methods tend to be highly efficient, where individual PPIs can be predicted in the fraction of a second. The first comprehensive predictions of intra-species interactomes for a number of organisms have been reported from sequence-based methods^11,12^. In particular, the Protein-protein Interaction Prediction Engine (PIPE; the latest version, MP-PIPE^11^, is denoted PIPE3 in this work for clarity) has been at the forefront of the these advances and has resulted in the elucidation of comprehensive interactomes for a large number of organisms including *Homo sapiens*, *Saccharomyces cerevisiae*, *Caenorhabditis elegans*, *Arabidopsis thaliana*, *Drosophila melanogaster*, *Mus musculus*, and *Glycine max*^3,11–17^.

## Increasing Scale and Complexity of Prediction Schemas

Comprehensive intra-species interactomes are highly useful to elucidate molecular biological processes within a given organism. Well-studied organisms with a large number of previously validated PPIs are amenable to these analyses, whereas under-studied organisms, with few PPIs from which to train a predictor, are problematic. Moreover, many under-studied species are of critical importance to human health and the elucidation of complete interactomes is necessary to investigate disease pathogenesis such as the Zika Virus^18^. We present a refined version of PIPE to address these challenges here.

Beyond providing insight into the cellular process within an organism, the application of PPI predictors to networks between organisms (*i.e*. inter-species) have led to the determination of disease pathogenesis^19–21^, development of novel pharmaceuticals^6,18^, and insights into the evolution of interactomes^22^. This work focuses on the computational challenges related to predicting extremely large interactomes and defining the best practices for combining inter- and cross-species prediction schemas to make predictions for organisms that might not normally be amenable to the generation of comprehensive interactomes.

### Cross-species predictions

**Figure 1:**
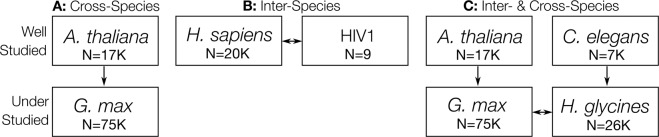
Inter- and Cross-Species Prediction Schemas. (**A**) Cross-species schema where the intra-species PPIs of a well-studied organism can be used as a proxy to predict the comprehensive interactome of another under-studied, but evolutionarily close, organism. (**B**) Typical inter-species prediction schema where PPIs within and/or between two well-studied organisms are used to predict the comprehensive interactome between the two organisms to identify novel putative PPIs. (**C**) Combination of the cross- and inter-species prediction schema where the intra-species PPIs of two well-studied organisms are used to train a model capable of predicting the comprehensive inter-species interactome of two under-studied organisms. N indicates the approximate size of the proteome.

Understudied organisms with very few experimentally validated PPIs, such as *G. max*, are not amenable to the study of comprehensive interactomes due to insufficient training data. To circumvent this, an evolutionarily similar, well-studied organism can serve as a *proxy*. That is, the experimentally validated intra-species PPIs from the *proxy* species are used to train the PPI predictor; predictions are then made for the proteome of the understudied *target* organism. Due to the limited availability of known *G. max* PPIs, we here use *Arabidopsis thaliana* as a well-studied and evolutionarily similar proxy to generate these cross-species predictions as depicted in Fig. 1A.

### Inter-species predictions

While PIPE has demonstrated preliminary successes when applied to inter-species prediction tasks^19^, this problem requires that the scoring function, which might account for the frequency of short, contiguous subsequences, is appropriate since proteome sizes can vary considerably between organisms, and we expect the number of similar subsequences to vary greatly as a result. This schema, depicted in Fig. 1B, requires that the prediction score of a PPI between two organisms normalizes the evidence from a given window in a protein by that window’s prevalence within its respective proteome. This work describes the *Similarity Weighted Score* which accounts for this.

### Predicted interactome size

Historically, PIPE has been applied to increasingly larger and more complex PPI prediction problems. Originally, PIPE was designed to predict a small number of pairs within a single species. It was progressively optimized to predict comprehensive interactomes that scale as the triangular number of a proteome of size *n*, $n(n+1)/2$. In the original organism, *S. cerevisiae*, with proteome size $n\approx 6,700$, this comprised a comprehensive interactome of 22 million uniquely predicted PPIs. Following considerable optimization through distributed computing, the application of PIPE to *H. sapiens*, with $n\approx 21,000$, comprised an interactome one greater order of magnitude: 220 million. Now, with a *G. max* proteome of $n\approx 75,700$, we operate at one greater order of magnitude still with 2.8 billion intra-species predictions. To rapidly compute such complex interactomes in a timely manner, the PIPE preprocessing data representation is adapted as described in the Methods section.

In combination, the appropriate organization of these increasingly complex prediction schemas promise to enable improved prediction of these highly useful entire interactomes. For example, although both the soybean legume, *G. max*, and the soybean cyst nematode (SCN), *Heterodera glycines*, are understudied organisms, we wish to identify actionable PPIs that might be disrupted to protect crop yields. Fortunately, *A. thaliana* and *C. elegans* are, respectively, evolutionarily similar and well-studied model organisms capable of serving as proxies to these two organisms of interest. This prediction schema, involving both inter- and cross-species PPI predictions is depicted in Fig. 1C.

Finally, high-throughput predictors have enabled the prediction of the comprehensive set of all possible interactions that has given rise to context: the ability to appraise a given PPI prediction relative to all possible interactions. Applying the Reciprocal Perspective for PPI (RP-PPI) cascaded machine learning layer to these data has led to significantly improved predictive performance in the face of extreme class imbalance^16^. As a meta-method applicable to any PPI predictor, here we looked to additionally validate RP-PPI for use in cross-species prediction schemas.

## Methods

The last decade has seen increased computational demand of PPI prediction tasks, both in scale and complexity^11,13,14,23^. The existing PIPE implementation, MP-PIPE2 published in^11^, which for the sake of clarity we denote as PIPE3 in this work, has been sufficient for these tasks. However, the comprehensive prediction of the *G. max* interactome necessitates scoring of 2,871,417,871 putative PPIs, which is more than a ten-fold increase in size over the comprehensive human interactome. We, here, introduce a more efficient version of PIPE, aptly named PIPE4, capable of handling prediction tasks involving the soybean legume in both inter- and cross-species prediction tasks. Briefly, the fundamental PIPE algorithm examines each sliding window of each protein to determine if they are sequence-similar to protein pairs known to physically interact. The preprocessing step tabulates which windows in proteins that are sequence-similar in order to accelerate the prediction step.

### Mathematical notation to describe algorithmic changes

We, here, clarify the notation used to describe the subsequent algorithmic changes to the PIPE algorithm. A bold symbol depicts a collection such as a vector, list, or set of elements. The subscripts are tailored to reflect inter-species applications and identify a certain protein within a given organism’s proteome. To simplify the notation, where appropriate, the subscripts are dropped. Unless otherwise stated, examples using one organism are implied for the other. Superscripts are used to identify the starting position of a certain contiguous subsequence within a given protein. Greek symbols (*e.g*. $\sigma ,\,\Phi ,\,\varphi ,\,\Gamma ,\,\gamma )$ are used to represent functions and, where appropriate, their capital notation (*e.g*. Φ, Γ) references the function while their lowercase notation (*e.g. φ, γ*) are used to conveniently indicate the size of the collection resulting from the application of that function. Following general convention, vertical bars, |·|, are used to represent the size of a given collection, and the overbar notation, $\bar{\cdot }$, is used for the average size of a collection. Finally, while the $\oplus$ symbol is conventionally used as the XOR function, we here use it as a concatentation operator.

**Figure 2:**
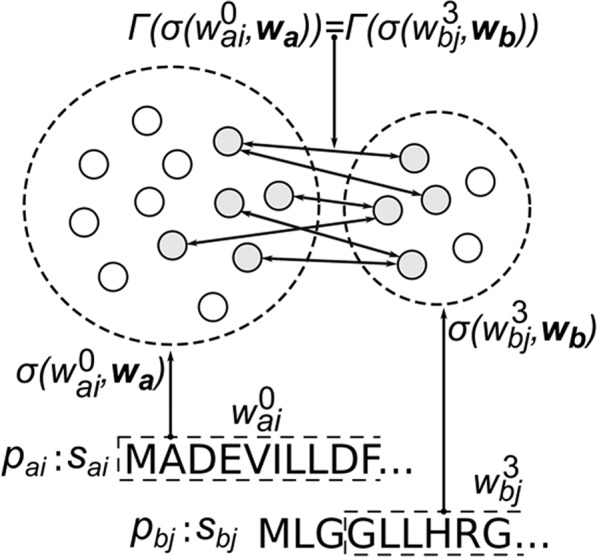
Visual Representation of Mathematical Notation. The comparison of two windows between two arbitrary proteins (${p}_{ai}$ and ${p}_{bj}$) yeilds two disjoint (inter-species) sets comprising proteins with similar subsequences to those windows. Grey proteins linked with arrows indicate known PPIs.

For an intuitive visualization of the notation used in the main text, Fig. 2 depicts an example comparison between two arbitrary proteins, *p*_*ai*_ and *p*_*bj*_, wherein the two windows are compared against all other windows within each organism’s proteome. In this case, the set of similar proteins are shown to be disjoint as would be expected in inter- and cross-species predictions; however, for intra-species prediction schemas, we can expect overlap in the sets of similar proteins. In this example case, the number of “hits” for this one pair of windows (the size of the intersection) is 6 and this value is normalized as described in the main text.

### Preprocessing protein sequences for window similarity

We first describe PIPE3′s preprocessing step, which identifies similar subsequences within a given proteome. We consider the proteome of organism *a* to be of size *n* and having both a list of protein names (represented as integer indices), ${{\boldsymbol{p}}}_{{\boldsymbol{a}}}=[{p}_{a1},{p}_{a2},\ldots ,{p}_{ai},\ldots ,{p}_{an}]$, and list of amino acid sequences, ***s***_***a***_ = [*s*_*a*1_, *s*_*a*2_, …, *s*_*ai*_, …, *s*_*an*_], with correspondence ${{\boldsymbol{p}}}_{{\boldsymbol{a}}}\to {{\boldsymbol{s}}}_{{\boldsymbol{a}}}$ to indicate that *p*_*ai*_ has sequence *s*_*ai*_. Similarly, organism *b* with proteome of size *m* has ${{\boldsymbol{p}}}_{{\boldsymbol{b}}}=[{p}_{b1},{p}_{b2},\ldots ,{p}_{bj},\ldots ,{p}_{bm}]$, and ${{\boldsymbol{s}}}_{{\boldsymbol{b}}}=[{s}_{b1},{s}_{b2},\ldots ,{s}_{bj},\ldots ,{s}_{bm}]$ with ${{\boldsymbol{p}}}_{{\boldsymbol{b}}}\to {{\boldsymbol{s}}}_{{\boldsymbol{b}}}$. We denote the amino acid sequence length of any *s* as *k* (*i.e*. ${\boldsymbol{s}}\to {\boldsymbol{k}}$ for *a*, *b*). A contiguous sequence of amino acids of length *l*, where *l* ≤ *k*, of *s* is denoted a “window”, *w*. Applying a sliding window of length *l* along sequence *s*_*ai*_, permitting overlap and shifting by one amino acid at a time, generates a list of windows: ${{\boldsymbol{w}}}_{{\boldsymbol{ai}}}=[{w}^{1},{w}^{2},\ldots ,{w}^{h},\ldots ,{w}^{(k-l)+1}]$. To clarify the notation, an example arbitrary window from protein *p*_*ai*_ with sequence *s*_*ai*_ and length *k*_*ai*_ in organism *a*’s proteome is given as ${w}_{ai}^{x}$ and analogously for protein *p*_*bj*_ as ${w}_{bj}^{y}$.

A similarity function, $\sigma ({w}_{ref},{w}_{query})$, is used to determine whether any two arbitrary windows in the proteome are deemed “similar”, as defined by a value *v* obtained via the PAM120 amino acid substitution matrix and similarity threshold, *τ*. The function takes as input two windows, where the first, *w*_*ref*_, is a *reference* window from one proteome and the second, *w*_*query*_, is the *query* window from another proteome. The function outputs the protein index of the protein containing *w*_*query*_ if the windows are similar. Furthermore, the function is defined to support vectors of windows as input (emphasized using boldface) and returns the corresponding set of proteins from the other proteome, where similar:

1 $$\sigma ({w}_{ai}^{x},{w}_{bj}^{y})=\left\{\begin{array}{cc}{p}_{bj}, & {\rm{i}}{\rm{f}}\,v\ge \tau \,{\rm{a}}{\rm{n}}{\rm{d}}\,{w}_{ref}\in {{w}}_{{a}}\\ {p}_{ai}, & {\rm{i}}{\rm{f}}\,v\ge \tau \,and\,{w}_{ref}\in {{w}}_{{b}}\\ \varnothing ,\, & {\rm{o}}{\rm{t}}{\rm{h}}{\rm{e}}{\rm{r}}{\rm{w}}{\rm{i}}{\rm{s}}{\rm{e}}\end{array},\right.$$

It is important to note that the window originating from proteome *a* returns a protein index from proteome *b*, and vice versa. Each window, ${w}_{ai}^{x}$ and ${w}_{bj}^{y}$, therefore has its own corresponding list of proteins, ${\boldsymbol{\Phi }}$ of length *φ*, having at least one window similar to it (*φ* ≥ 1). With respective lengths ${\varphi }_{ai}^{x}$ and ${\varphi }_{bj}^{y}$, these lists are denoted ${{\boldsymbol{\Phi }}}_{{\boldsymbol{ai}}}^{{\boldsymbol{x}}}=[{p}_{bj},{p}_{bj+1},\ldots ,{p}_{b\varphi }]$ and ${{\boldsymbol{\Phi }}}_{{\boldsymbol{bj}}}^{{\boldsymbol{y}}}=[{p}_{ai},{p}_{ai+1},\ldots ,{p}_{a\varphi }]$. To efficiently store these window similarities, a database file, *d*, for each protein is written as a ragged array of unsigned integers representing the indices of proteins found to be similar to its windows. The data for an arbitrary protein *p* is written with the following format:

2 $${d}_{p}:k\oplus {\varphi }^{1}\oplus {\Phi }^{1}\oplus \ldots \oplus {\varphi }^{(k-l)+1}\oplus {\Phi }^{(k-l)+1}$$

This preprocessing step is run for all proteins in each organism’s proteome to produce a data structure enabling constant time access during the prediction of the complete interactome. Analysis of the computational runtime complexity of a single pair of proteins (where the overbar denotes the average) of this preprocessing step yields:

3 $$O(\bar{{k}_{a}}\,\bar{{k}_{b}}\,\bar{\varphi })$$

Here, $\overline{{k}_{a}}$ and $\overline{{k}_{b}}$, are the average sequence length from species *a* and *b* respectively. Preprocessing is only run once and, having a runtime several orders of magnitude less than the remainder of the PIPE algorithm, it is accepted as a flat start-up cost and thus negligible when analysing the remaining PIPE runtime.

**Algorithm 1:**
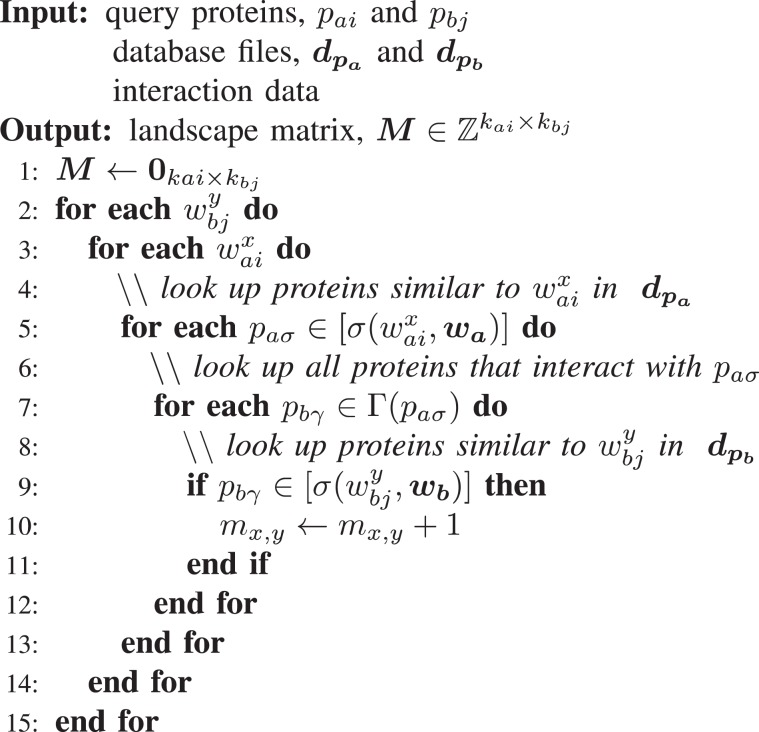
PIPE Landscape Generation Algorithm.

### The landscape generation algorithm

For any pair of proteins, *p*_*ai*_ and *p*_*bj*_, we generate a score representative of their likelihood of physical interaction. A sufficiently large score, exceeding some globally defined decision threshold, results in a positive classification. The score for any pair is the result of applying an aggregation function to a landscape, defined as matrix ${\bf{M}}\in {{\mathbb{Z}}}^{{k}_{ai}\times {k}_{bj}}$. The landscape generation algorithm can be summarized as follows: *To determine the landscape value at position (x, y), examine the pair of windows*, ${w}_{ai}^{x},{w}_{bj}^{y}$, *and count the number of known interactions involving a pair of proteins with similar windows*.

Algorithm 1 illustrates an implementation of this process and Figure 2 serves as an additional pictoral depiction. For clarity, we define the interactor function, $\Gamma$(·), which takes as input a protein index, *p*, and returns a list of length *γ* containing all proteins participating in a known interaction with *p*. For example:

4 $$\varGamma ({p}_{ai})=\left\{\begin{array}{ll} \varnothing & {\rm{if}}\,\gamma =0\\{[{p}_{by}]}, & {\rm{if}}\,\gamma =1\\{[{p}_{bj},\ldots ,{p}_{by}]}, & {\rm{if}}\,\gamma  > 1\end{array}\right.,$$

Analysis of the computational complexity of the PIPE landscape generation algorithm, *O*_*L*_, yields:

5 $$O{\textstyle (}\bar{{k}_{a}}\,\bar{{k}_{b}}\,\bar{\varphi \,}\bar{\gamma }{\textstyle )}$$

The terms $\overline{{k}_{a}}$ and $\overline{{k}_{b}}$, through correspondence, arise from lines 2 and 3 of Algorithm 1, where each pair of windows in proteins *p*_*ai*_ and *p*_*bj*_ are considered. Since these windows overlap, the number of windows is proportional to the length of each protein. Lines 5 and 7 denote the average number of similar proteins to a given window, $\bar{\varphi }$, and the average number of interactions for a given protein, $\bar{\gamma }$, respectively. Note that the subscripts are dropped to emphasize that the averaging occurs over both proteomes. Lines 9 and 10 comprise *O*(1) time set membership operations and matrix increments. With the landscape for a given PPI computed, the final score is obtained with the application of an aggregation function. Of particular importance to this function, when leveraged for inter-species predictions, is the normalization of windows by their prevalence in each respective organism’s proteome.

### The similarity weighted score

The Similarity Weighted Score (SW-score) was originally proposed as part of Catalin Patulea’s seminal 2011 work^24^. Having initially led to significant improvement over the traditional PIPE Score of the time, it has renewed implication when used for inter-species and cross-species predictions. The Similarity Weighting function, $\lambda (x,y)$, takes as input the coordinates of a cell, with indices *x* and *y*, in the landscape matrix and has the form:

6 $$\lambda (x,y)=|\sigma ({w}_{ai}^{x},{{\bf{w}}}_{{\bf{a}}})||\sigma ({w}_{bj}^{y},{{\bf{w}}}_{{\bf{b}}})|$$

The SW-score is an aggregated value of the average of the landscape following the application of $\lambda (x,y)$ to all cells in the landscape. The function normalizes the height of the landscape at a point by counting the number of possible interactions arising if every pair of similar proteins were to interact. This suppresses the effect of highly prevalent windows that are not associated with interactions and amplifies the effect of windows that are relatively rare, yet are frequently occurring in known interactions. Consequential to inter-species applications, the set of all windows in organism *a*, ${{\bf{w}}}_{{\bf{a}}}$, can differ greatly from the set of organism *b*, ${{\bf{w}}}_{{\bf{b}}}$; whereas, for intra-species applications, by definition ${{\bf{w}}}_{{\bf{a}}}={{\bf{w}}}_{{\bf{b}}}$.

Furthermore, when applied to cross-species predictions, training data may be pooled from multiple organisms, in which case this normalization requires modification due to the potentially dramatic differences in the number of training examples from each species. In this work, we consider using *A. thaliana* as a well-studied proxy to make cross-species predictions on behalf of *G. max*, for which very limited training data is available. Similarly, we use *C. elegans* as a proxy for *H. glycines* to ultimately predict the inter-species interactome between the latter two. Naïve application of Eq. (6) to this PPI prediction schema, using subscripts *A*, *G*, *C*, and *H* to respectively denote the proteomes of *A. thaliana*, *G. max*, *C*. elegans, and *H. glycines*, we obtain:

7 $$\begin{array}{c}\lambda (x,y)={\textstyle (}|\sigma ({w}_{Ai}^{x},{{\bf{w}}}_{{\bf{A}}})|+|\sigma ({w}_{Gi}^{x},{{\bf{w}}}_{{\bf{G}}})|+|\sigma ({w}_{Ci}^{x},{{\bf{w}}}_{{\bf{C}}})|+|\sigma ({w}_{Hi}^{x},{{\bf{w}}}_{{\bf{H}}})|{\textstyle )}\\ \,\,{\textstyle \,(}|\sigma ({w}_{Aj}^{y},{{\bf{w}}}_{{\bf{A}}})|+|\sigma ({w}_{Gj}^{y},{{\bf{w}}}_{{\bf{G}}})|+|\sigma ({w}_{Cj}^{y},{{\bf{w}}}_{{\bf{C}}})|+|\sigma ({w}_{Hj}^{y},{{\bf{w}}}_{{\bf{H}}})|{\textstyle )}\end{array}$$

Simplifying the frequency of window ${w}_{Ai}^{x}$ in the *A. thaliana* proteome as ${f}_{Ai}^{x}=|\sigma ({w}_{Ai}^{x},{{\bf{w}}}_{{\bf{A}}})|$, Eq. (7) becomes:

8 $$\lambda (x,y)=({f}_{Ai}^{x}+{f}_{Gi}^{x}+{f}_{Ci}^{x}+{f}_{Hi}^{x})({f}_{Aj}^{y}+{f}_{Gj}^{y}+{f}_{Cj}^{y}+{f}_{Hj}^{y})$$

Formulated in this way, Eq. (8) implies that there are possible interactions between *A.thaliana* – *G.max*, *A. thaliana* – *C. elegans, A. thaliana* – *H. glycines*, *C. elegans* – *H. glycines*, and *C. elegans* – *G. max*, and normalizes it as such. While *in vitro* interactions are certainly possible for any combination of these organisms, no such interactions are included in the training data which unfairly penalizes any windows that are similar to windows in multiple species. With the incorporation of additional species with similar proteins, this effect becomes increasingly pronounced. Furthermore, a window is normalized not only by how frequently it appears in the training data, but also in the target organism proteome for which very little or no interaction data is known. Consequently, the normalization factor does not reflect the true number of possible interactions among similar proteins in the training data. We here propose a cross-species variant of Eq. (8), which normalizes window frequency only in species for which there are available PPI training data:

9 $$\lambda (x,y)=({f}_{Ai}^{x}+{f}_{Ci}^{x})({f}_{Aj}^{y}+{f}_{Cj}^{y})$$

A number of experiments validating this modification to the SW-score are described in Supplementary File. Irrespective of its form, loading the database files into RAM enables constant-time lookup to similar windows in respective proteomes. The complexity for computing the SW-score for a given putative PPI between *p*_*a*_ and *p*_*b*_ (say between *G. max* and *H. glycines*) applies $\lambda (x,y)$ to each cell and the subsequent aggregation yield an average runtime of:

10 $$O{\textstyle (}\bar{{k}_{a}}\,\bar{{k}_{b}}{\textstyle )}$$

### The modified PIPE algorithm

Considering the end-to-end time complexity of PIPE, we obtain:

11 $${O}_{PIPE3}=O{\textstyle (}\bar{{k}_{a}}\,\bar{{k}_{b}}\,\bar{\varphi }{\textstyle )}+\frac{mn}{2}(O{\textstyle (}\bar{{k}_{a}}\,\bar{{k}_{b}}\,\bar{\varphi \,}\bar{\gamma }{\textstyle )}+O{\textstyle (}\bar{{k}_{a}}\,\bar{{k}_{b}}{\textstyle )}{\textstyle )}$$

The one-time precomputation term is negligible in size in comparison to the landscape and aggregation function terms which must be computed for all *mn*/2 possible interactions. Due to the current inescapability of the terms $\overline{{k}_{a}}$ and $\overline{{k}_{b}}$ from the necessity to compare all pairwise windows, the only free terms for optimization are $\bar{\varphi }$ (average number of similar proteins to a given window) and $\bar{\gamma }$ (average number of interactions for a given protein). Analysis of Algorithm 1 indicates that lines 5–10 compute the height of the landscape at a single point and can be reformulated as: *Given windows* ${w}_{ai}^{x}$ *and* ${w}_{bj}^{y}$*, count the number of proteins in the intersection of* $\Gamma (\sigma ({w}_{ai}^{x},{{\bf{w}}}_{{\bf{a}}}))$ *and* $\sigma ({w}_{bj}^{y},\,{{\bf{w}}}_{{\bf{b}}})$:

12 $${m}_{x,y}={\textstyle |}\Gamma (\sigma ({{\rm{w}}}_{{\rm{a}}{\rm{i}}}^{{\rm{x}}},\,{{\bf{w}}}_{{\bf{a}}})){\cap }^{}\sigma ({{\rm{w}}}_{{\rm{b}}{\rm{j}}}^{{\rm{y}}},\,{{\bf{w}}}_{{\bf{b}}}){\textstyle |}$$

The first set, $\Gamma (\sigma ({w}_{ai}^{x},\,{{\bf{w}}}_{{\bf{a}}}))$, comprise proteins from species *b* that interact with the proteins from species *a* containing one or more windows similar to the window ${w}_{ai}^{x}$. The second set, $\sigma ({w}_{bj}^{y},{{\bf{w}}}_{{\bf{b}}})$, comprise proteins that contain one or more windows similar to the window ${w}_{bj}^{y}$. A protein at this intersection represents a known interaction between a protein similar to ${w}_{ai}^{x}$ and a protein similar to ${w}_{bj}^{y}$. The runtime to perform this set intersection with PIPE3 (Algorithm 1) is:

13 $$O{\textstyle (}\Gamma {\textstyle (}\sigma ({w}_{ai}^{x},{{\bf{w}}}_{{\bf{a}}}){\textstyle )}{\textstyle )}=O{\textstyle (}\bar{\varphi }\,\bar{\gamma }{\textstyle )}$$

The probability, ${\rm{p}}$(·), of any one protein, ${p}_{b\ast }$, residing in this intersection is given as:

14 $${\rm{p}}{\textstyle (}{p}_{b\ast }\in \sigma ({w}_{bj}^{y},{{\bf{w}}}_{{\bf{b}}}){\textstyle )}=\frac{|\sigma ({w}_{bj}^{y},{{\bf{w}}}_{{\bf{b}}})|}{m+n}\approx \frac{\bar{\varphi }}{m+n}$$

Where, by definition $\bar{\varphi }\le m+n$ and generally $\bar{\varphi }\ll m+n$. Therefore, while looping through each protein in $\Gamma {\textstyle (}\sigma ({w}_{ai}^{x},\,{{\bf{w}}}_{{\bf{a}}}){\textstyle )}$, only very rarely will it occur in the intersection. These costly set intersections are unavoidable for any given pair of sets. Moreover, they must be repeated $1/2(mn\,\overline{{k}_{a}}\,\overline{{k}_{b}})$ times. To circumvent this costly intersection computation, an alternate preprocessing data representation is proposed.

With the intent of directly precomputing these set intersections (such that membership checks of this intersection are never false), we look to first generate a hash table data representation having a key:value pair where the key comprises each potential protein of the set intersection, ${p}_{\psi }$, while its associated value comprises a list of the indices of the sliding windows where this protein occurs within each input protein. We define the indices retrieval function, $\Psi$(·), which takes as input a protein and returns a list of all the indices of the similar windows occurring within a set of proteins (note the boldface):

15 $$\Psi ({p}_{\psi })=\left\{\begin{array}{cc}x, & {\rm{i}}{\rm{f}}\,{p}_{\psi }\in \Gamma {\textstyle (}\sigma ({w}_{ai}^{x},{{\bf{w}}}_{{\bf{a}}}){\textstyle )}\,{\rm{\forall }}\,i:1,\ldots ,n\\ y, & {\rm{i}}{\rm{f}}\,{p}_{\psi }\in \sigma ({w}_{bj}^{y},{{\bf{w}}}_{{\bf{b}}})\,{\rm{\forall }}\,j:1,\ldots ,m\,\\ \varnothing , & {\rm{o}}{\rm{t}}{\rm{h}}{\rm{e}}{\rm{r}}{\rm{w}}{\rm{i}}{\rm{s}}{\rm{e}}\,\end{array},\right.$$

Instead of storing the set of proteins ${\rm{\sigma }}({{\rm{w}}}_{{\rm{bj}}}^{{\rm{y}}},\,{{\bf{w}}}_{{\bf{b}}})$ for each window location *y* in protein *p*_*bj*_, we instead store $\Psi ({{\boldsymbol{p}}}_{{\boldsymbol{b}}})$ which is the set of all locations ${\boldsymbol{y}}$ for which the set $\sigma ({w}_{bj}^{y},\,{{\bf{w}}}_{{\bf{b}}})$ contains ${p}_{\psi }$. Similarly, we store $\Psi ({{\boldsymbol{p}}}_{{\boldsymbol{a}}})$ which is the set of all locations ${\boldsymbol{x}}$ for which the set $\Gamma {\textstyle (}\sigma ({w}_{ai}^{x},\,{{\bf{w}}}_{{\bf{a}}}){\textstyle )}$ contain ${p}_{\psi }$. In essence, rather than list all proteins similar to a given window, we list all windows similar to a given protein, enabling the direct acquisition of common subsets (and thereby an interaction landscape) for every pair of windows by examining each ${p}_{\psi }$ and incrementing the landscape for every pair of windows between $\Psi ({{\boldsymbol{p}}}_{{\boldsymbol{a}}})$ and $\Psi ({{\boldsymbol{p}}}_{{\boldsymbol{b}}})$. A desirable consequence is that we never perform a membership check which returns false and the expected reduction in computational landscape generation time is a factor of:

16 $$\frac{{O}_{PIPE4}}{{O}_{PIPE3}}=\frac{\bar{\varphi }}{m+n}$$

The speedup of PIPE4 determined here is a notable increase over the PIPE3 algorithm^11^, which had previously improved over the former PIPE2 algorithm^23^. Implementation of these modifications requires two new database file representations. To simplify the notation, we define a list of indices as ${{\boldsymbol{\Psi }}}_{{\boldsymbol{ai}}}=\Psi ({p}_{ai})$ and having length $|{{\boldsymbol{\Psi }}}_{{\boldsymbol{ai}}}|$. The first database file, denoted ${d^{\prime} }_{w}$, follows the same format as the original preprocessed version in (2), only instead of storing the proteins similar to each window of the database protein, we store the similar windows for each protein in the proteome. The format of ${d^{\prime} }_{w}$ is as follows:

17 $${d^{\prime} }_{w}:m\oplus |{{\boldsymbol{\Psi }}}_{{\boldsymbol{b}}1}|\oplus {{\boldsymbol{\Psi }}}_{{\bf{b}}1}\oplus \ldots \oplus |{{\boldsymbol{\Psi }}}_{{\boldsymbol{bm}}}|\oplus {{\boldsymbol{\Psi }}}_{{\boldsymbol{bm}}}$$

The second database file, denoted ${d}_{w}^{^{\prime\prime} }$, for a given protein, *p*, lists the window locations in *p* that are similar to a protein that interacts with each ${p}_{\psi }$:

18 $${d}_{w}^{^{\prime\prime} }:n\oplus |{{\boldsymbol{\Psi }}}_{{\boldsymbol{a}}1}|\oplus {{\boldsymbol{\Psi }}}_{{\bf{a}}1}\oplus \ldots \oplus |{{\boldsymbol{\Psi }}}_{{\boldsymbol{an}}}|\oplus {{\boldsymbol{\Psi }}}_{{\boldsymbol{an}}}$$

The modified PIPE landscape generation algorithm leveraging these representations is detailed in Algorithm 2. Analysis of the computational complexity of the modified landscape generation algorithm, ${O^{\prime} }_{L}$, yields:

19 $$O{\textstyle (}|{\bar{{\boldsymbol{\Psi }}}}_{{\boldsymbol{a}}}|\,|{\bar{{\boldsymbol{\Psi }}}}_{{\boldsymbol{b}}}|{\textstyle )}$$

Where $|{\bar{{\boldsymbol{\Psi }}}}_{{\boldsymbol{a}}}|$ is the average number of similar windows for a protein in a ${d}_{w}^{^{\prime} }$ database file and $|{\bar{{\boldsymbol{\Psi }}}}_{{\boldsymbol{b}}}|$ is the average number of similar windows for a protein in a ${d}_{w}^{^{\prime\prime} }$ database file. These terms can be further broken down into:

20 $$|{\bar{{\boldsymbol{\Psi }}}}_{{\boldsymbol{a}}}|=\left(\frac{\bar{{k}_{a}}\,\bar{\varphi }}{m+n}\right)\bar{\gamma }\,$$

21 $$|{\bar{{\boldsymbol{\Psi }}}}_{{\boldsymbol{b}}}|=\frac{\bar{{k}_{b}}\,\bar{\varphi }}{m+n}$$

Which, when substituted into (19), gives:

22 $$O\left(\frac{\bar{{k}_{a}}\,\bar{{k}_{b}}\,\bar{{\varphi }^{2}}\,\bar{\gamma }}{m+n}\right)$$

Incorporating the original landscape generation complexity, *O*_*L*_, we determine that the modified landscape generation complexity, ${O^{\prime} }_{L}$, is:

23 $$O\left({O}_{L}\times \frac{\bar{\varphi }}{m+n}\right)$$

Hence, we confirm the assertions from (14) and (16). Since $\bar{\varphi }\ll m+n$, generally, the new landscape generation algorithm is expected to be considerably faster at a rate proportional to the size of the proteomes of the organisms involved in the PPI prediction schema. Additional details of the experimental validation of the modified PIPE algorithm are available in the Supplementary File.

**Algorithm 2:**
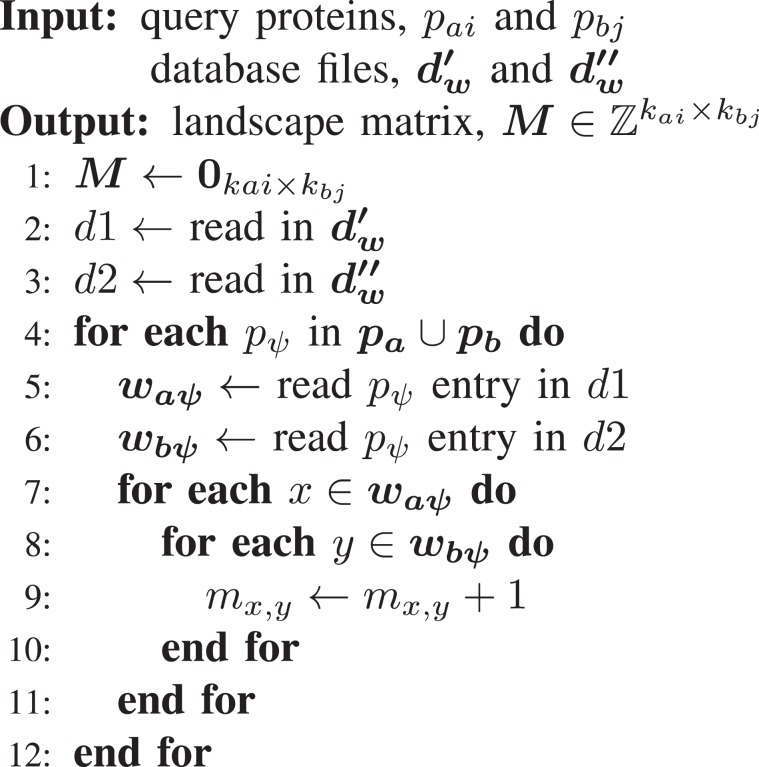
Modified PIPE Landscape Algorithm.

### Predicting the comprehensive soybean, human-HIV1, and soy-SCN interactomes

To predict comprehensive interactomes, we require known PPI data and sequence data. All PPI training data were obtained from BioGRID, where interaction data were filtered to only include physical PPIs. These data were assembled in accordance to the three schemas represented in Fig. 1: the *A. thaliana* intra-species interactions were obtained for Fig. 1A, the *H. sapiens*-HIV1 inter-species (only) interactions were obtained for Fig. 1B, and both *A.thaliana* and *C. elegans* intra-species interactions were obtained for Fig. 1C. Any duplicate interactions were removed. The amino acid sequences for the *H. sapiens, A. thaliana, C. elegans*, and HIV1 proteomes were obtained from the UniProt database selecting only the manually annotated and reviewed Swiss-Prot proteins. In the event that a known interaction from BioGRID did not correspond to a Swiss-Prot protein, the sequence was instead taken from the larger TrEMBL UniProt database, which comprises automatically annotated and reviewed proteins. The *G*. max and *H. glycines* sequences were each obtained from SoyBase and SCNbase respectively. The three interactomes defined in Fig. 1 were then predicted using the PIPE4 algorithm as described above.

### Dataset preparation for multi-organism inter- and cross-species experiments

A high-quality dataset of previously known interacting PPIs was required for each of the organisms considered in this work. For each organism, we downloaded from BioGRID^25^ those intra-species PPI data that comprise physical PPIs (eliminating genetic interactions, co-localization, or functional associations). Intra-species PPIs were selected to simulate the use of one organism’s intra-species PPIs to predict another organism’s intra-species PPIs. Duplicate interactions were removed from the data and finally filtered to exclude species with ten or fewer PPIs. The seventeen species that met this criterion are tabulated with their dataset compositions in Supplementary Table S4. The amino acid sequences of every protein within these PPI datasets was downloaded from the UniProt database^26^. The Swiss-Prot database of manually annotated and reviewed proteins was used, and only when a known interaction from BioGRID did not correspond to a Swiss-Prot protein, did we extract the sequence from the larger TrEMBL UniProt database (comprising automatically annotated and reviewed proteins).

### Cross-species validation experiments

A preliminary demonstration of PIPE’s ability to complete cross-species PPI prediction was presented in^27^, wherein the known PPIs from *S. cerevisiae* could be used to predict *H. sapiens* PPIs and vice-versa. While it was shown that the predictions improved over random chance, general best practices for making cross-species predictions were not examined further. The central hypothesis of this work considered the performance rank to be inversely correlated with evolutionary distance rank; *i.e*. it is expected that more closely related species would perform better in cross-species prediction tasks due to evolutionary conservation of protein sequence, function, and structure.

One-to-Many Cross-Species predictions use the trained model of one source organism to make predictions for other target organisms to examine the relationship of evolutionary distance with classification performance using area under the precision-recall curve (AUPRC) and precision at 25% recall (Pr@25Re) metric. Many-to-One Cross-Species predictions use the trained model of multiple pooled organisms to make predictions for a single target organism. For these latter experiments, Kendall’s Tau-b and Spearman rank correlation tests were used to examine whether relative evolutionary distance correlated to predictive performance. The training data for each organisms was obtained from BioGRID and the protein sequences from UniProt; their specific composition is tabulated in Supplementary Table S4.

### Evolutionary distance relation to classification performance

**Figure 3:**
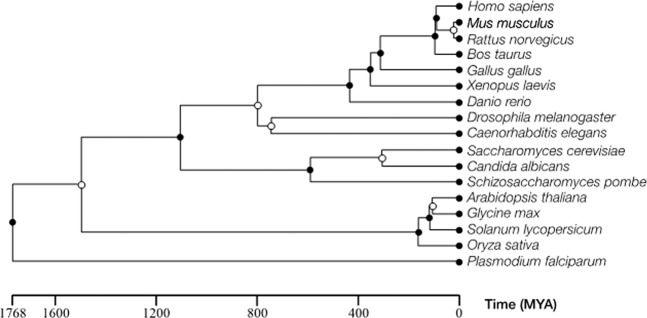
Estimated Evolutionary Divergence Timeline.

A central hypothesis to this work is that evolutionary distance will influence predictive performance such that more closely related organisms will produce improved cross-species PPI prediction. This was tested by estimating the evolutionary distance between each organism using a phylogenetic tree and correlating that distance with the resulting predictive performance for each condition (Fig. 3), however only the eight species amenable to random subsampling of 2000 training PPIs (to control for amount of training data) were considered. Of particular note, certain species may be evolutionarily equidistant to others such as *H. sapiens* with both of *M. musculus* and *R. norvegicus*. In this case, we expect the resulting rank to be the same. For example, when predicting *M. musculus* PPIs, the highest performance would be expected when training with *M. musculus* PPIs, followed by training with *R. norvegicus* PPIs, followed by *H. sapiens* PPIs and so forth. The comprehensive interactomes for all pairwise combinations of these eight organisms were predicted. Using a random subsample of 100,000 non-interacting pairs as the negative set, ROC curves were generated for all combinations and repeated 20 times (to control for the random sampling procedure); the average ROC curve was reported. Correlation between evolutionary distance and performance was measured using Spearman and Kendall’s Tau-b rank correlation tests.

Kendall’s Tau-b rank correlation test and the Spearman rank correlation test were both used to test the correlation between evolutionary distance and performance (both AUPRC and Pr@25Re) with each training species. We selected a class imbalance of 10:1 (negative:positive) when computing prevalence-corrected precision for the AUPRC metric. The prevalence-corrected precision (PCPR) is defined as:

24 $$PCPR=\frac{Sn}{Sn+r(1-Sp)},\,{\rm{where}}\,{\rm{r}}=10\,{\rm{for}}\,1:10\,{\rm{imbalance}}$$

which conveniently enforces the selected class-imbalance, regardless of the number of positive or negative test samples. The class imbalance can influence the relative ordering of the AUPRC metric, but has no bearing on the Pr@25Re metric. Kendall’s Tau-b rank correlation equation has the form:

25 $${\tau }_{B}=\frac{{n}_{c}-{n}_{d}}{\sqrt{({n}_{0}-{n}_{1})({n}_{0}-{n}_{2})}}$$

where *n*_*c*_ and *n*_*d*_ are the number of concordant and discordant pairs of rank. These respectively count the number of times when the expected and actual ranks are homogenously larger or smaller in one row (concordant) and the number of times they are not (discordant). The divisor corrects for the number of non-duplicate rank pairs, where *n*_1_ and *n*_2_ are the number of duplicate ranks for each column and ${n}_{0}=n(n-1)/2$. Here, we adapted this correlation coefficient by counting the *n*_*c*_ and *n*_*d*_ for each test species independently and then summing the results for a single Tau-b coefficient which encompasses every pair of the training and test species. Since this adaptation may invalidate the assumptions of a conventional *p*-value estimation of the Tau-b test (specifically multiple-testing), we corroborate our findings with the permutation-based estimation, described below.

Evaluation of statistical significance was achieved using two independent methods. The first (implemented in the R language) approximated the Tau-b and Spearman distributions. The second generated the distribution through permutation tests where the evolutionary ranks are shuffled randomly over multiple iterations and the correlation coefficients calculated in each instance and the resulting *p*-value comprises the percentage of shuffled ranks producing a correlation coefficient equal to or more extreme than the experimentally observed value. An example PR curve for *H. sapiens* is depicted in Supplementary Fig. S5 and suggests that those organisms more closely related to the test species provide higher cross-species prediction accuracy. The summary of the statistical tests for both the AUPRC and Pr@25Re experiments are listed in Supplementary Tables S7 and S8, respectively.

### Inter-species validation experiments

The *H. sapiens*-HIV1 inter-species schema involves two well-studied organisms with sufficient inter-species training samples to predict the comprehensive interactome. While the majority of these PPIs result from *in vitro* experiments, the comprehensive inter-species interactome is valuable for providing insights into disease pathogenesis and the discovery of putative drug targets. Given the large evolutionary distance between Human and HIV1, this inter-species schema is ideally suited to evaluating the performance of PIPE4 over its previous version. Moreover, the set of predictions promises to be highly valuable to the design, development, and testing of human therapeutics. Intra-species PPIs for both *H. sapiens* and HIV1 were pooled together to create the training dataset. The previously published inter-species PPIs were then used to evaluate the performance of the PPI predictors. The negative dataset comprised a randomly sampled set of PPIs equivalent in size to the set of known positives. The performance of the new PIPE4 scoring function is compared to the SPRINT PPI-predictor, the SPPS predictor, the former PIPE3 PIPE-Score, and original Similarity Weighted score using a Prevalence-corrected Precision-Recall (PR) curve. Given that there is no consensus to the prevalence of negative PPIs within the Human-HIV interactome, we opt to use 10:1 following from previous estimates in other species and for consistency with other experiments presented. See section “Comparison to the State of the Art” for results. The comprehensive set of predictions between Human-HIV1 were deposited in the Dataverse for use by the broader community^28^.

### Reciprocal perspective

The RP-PPI meta-method, as described in^16^, is a cascaded machine learning layer which leverages context-based features to improve the classification performance of PPI predictors. We applied it to each of the interactomes generated in this work.

To validate the RP-PPI method for cross-species predictions, the cascaded classifier trained and evaluated on one species was used to make predictions for another using only the Rank- and Fold-Type features, as described in^16^; Score-type features were excluded to control for score biases between organisms. This was performed for all combinations of intra-species datasets over five organisms: *H. sapiens*, *M. musculus*, *C. elegans*, *S. cerevisiae*, *A. thaliana*. The average increase in AUPRC, following 1,000 bootstrap iterations, over the baseline PIPE4 cross-species performance was summarized as a heatmap (see section “Reciprocal Perspective for Inter- and Cross-Species Predictions”).

### Comparing PIPE4 with the state-of-the-Art PPI predictors

Finally, we compare the improved PIPE4 method against comparable PPI prediction methods with respect to computational speed, predictive performance, and requisite computational resources (e.g. amount of RAM required). While a broad suite of methods exists, we restrict our comparison to only those methods capable of predicting all possible pairs of interaction within or between organisms and are thus available to leverage the RP-PPI method that leads to improved predictive performance. Following previous benchmark comparisons^10^, we considered the method of Guo *et al*.^29^, Martin *et al*.^30^, a more recent SVM-based method based on Shen *et al*.^31^ denoted “Sequence-based Protein Partners Search” (SPPS)^32^, and the recently released SPRINT method^33^.

The Guo predictor makes use of a support vector machine (SVM) that leverages a feature vector for a protein sequence comprising the auto-correlation values of seven physicochemical properties which are concatenated for a given protein pair. The Martin predictor relies on feature vectors encoding sequence information within the product of signatures that are defined to be a culled set of subsequences; these are then used in an SVM to classify PPIs. The SPPS method is a more recent sequenced-based SVM predictor of PPIs wherein the encoded PPI information from the protein sequences is projected into a vector space of frequencies of conjoint triads (physiochemical properties of an amino acid and its vicinal amino acids are examined as a unit); these are then amenable for use in an SVM predictor. The SPRINT method uses spaced-seeds to encode similarity within subsequences and then processes these elements to eliminate those that occur too often to be involved in interactions.

Based upon the benchmark results determined in the SPRINT paper, where their performance was compared to both the Martin and Guo methods, the time and memory requirements (>2 weeks compute time, and/or >256GB of memory) of these two methods were too extreme for further consideration. Neither of these methods is capable of predicting the entire human interactome (~203 million possible pairs) in a reasonable time frame, and by extension, are unable to predict the entire soybean interactome (~2.8 billion possible pairs). Moreover, the Guo and Martin methods were previously compared with the PIPE2 method, finding the ancestral version of PIPE to exhibit superior performance^10^. By transitive property, it is superfluous to compare PIPE4 to Martin and Guo. For these reasons, for the large-scale comprehensive interactome prediction tasks (e.g. soy vs. SCN), we restrict our comparison to the only other method reported capable of predicting comprehensive interactomes, SPRINT. However, for a more modestly-sized inter-species prediction task (Human-HIV1), we compared the PIPE3, SPRINT, and SPPS predictor against PIPE4.

These experiments were conducted on the Agriculture and Agri-Foods’ high-performance cluster, denoted BioCluster, comprising 9 Dell PowerEdge R930’s in Dual Socket configuration, Intel(R) Xeon(R) CPU E7-8870 v4 at 2.10 GHz, 1TB of RAM at 1600 MHz, 1.7 TB SATA SSD. To fairly compare the runtime of each method, each predictor was allocated 20 parallel threads, each with 256GB of RAM. See section “Comparison to the State of the Art” for results.

## Results

With the need for increasingly large-scale and complex PPI prediction schemas, contemporary algorithms must be adapted to efficiently predict comprehensive interactomes. Here, the next iteration of the PIPE algorithm is adapted for application to inter- and cross-species predictions with improvements in computational time complexity and predictive performance. To our knowledge, PIPE4 is the first PPI predictor developed specifically with complex PPI prediction schemas in mind. In addition to its computational efficiency and PPI prediction accuracy being competitive with the state-of-the-art, PIPE can additionally propose the putative site of interaction using the PIPE-Sites algorithm, previously described.

The comparative study by Park was one of the first evaluations of PPI predictive performance for intra-, inter-, and cross-species schemas. While only Human and Yeast were considered at the time, the PIPE2 algorithm that was used in the study was demonstrated to outperform the competing methods when considering precision and recall^10^. The subsequent PIPE3^11^ sought to massively scale the PIPE algorithm for use in comprehensive prediction tasks (predicting proteome-wide interaction networks) and now in its fourth iteration, the PIPE4 algorithm is adapted to inter-species and cross-species prediction schemas. Here, we look to build extensively upon the previous findings of Park by taking a more thorough examination of the factors involved in these complex schemas.

### PIPE4 speedup experiments

PPI prediction is embarrassingly parallel and therefore the total algorithmic runtime is a function of the time required to calculate the score for an individual PPI. Written in the C language and using MPI and OpenMP for parallelization, the PIPE4 algorithm was run on a medium-sized local computing cluster. Comprising 18 compute nodes, each contains a 100 GB SSD, 32 GB of RAM, and an Intel Core i7-3770 8-core processor at 3.40 GHz. Comparing the previous PIPE3 with the current PIPE4 methods on the same benchmark datasets (intra-species predictions to appropriately appraise each version) we observe findings congruent with the derived asymptotic complexity (Table 1). That is, the relationship between the speedup and proteome size is linear; the larger the predicted proteomes, the greater the speedup.

**Table 1:** Intra-Species Benchmark Results on a Medium Sized Cluster.

Benchmark Measure	H. sapiens		A. thaliana		S. cerevisiae	
	PIPE4	PIPE3	PIPE4	PIPE3	PIPE4	PIPE3
Database Size (GB)	18	1.6	5.6	0.7	1.9	0.2
Database Processing (s)	3191	3194	1358	1325	263	255
Predicted Positive Pair (s)	0.0155	0.7700	0.0061	0.1103	0.0113	0.1280
Predicted Negative Pair (s)	0.0084	0.4447	0.0049	0.0615	0.0054	0.0448
All-to-All Prediction (h)	3.3	175.9	1.4	17.6	0.2	2.0
Landscape Generation (s)	0.0056	0.4405	0.0022	0.0586	0.0029	0.0427
Total Speedup (~x)	**53.2×**		**12.5×**		**8.4×**	
Landscape Generation (~x)	**79.2×**		**26.4×**		**14.8×**	
Proteome Size, n	20,236		17,226		6,721	

All experiments run using 18 nodes with 8 threads/node.

The computational space-time trade-off resulted in a substantial increase in database file size. The corresponding data structure must fit into available RAM; however, we note that even for the largest proteome, this does not exceed the modest capacity of the machines used. Since the vast majority of use cases are expected to be performed on proteomes equal to or smaller than the human proteome, the increase in database size is an acceptable trade-off for the improvements in computational time. Moreover, when compared to contemporary sequence-based methods, such as SPRINT, the PIPE4 algorithm now makes predictions within an order of magnitude using comparable infrastructure.

### Evolutionary distance relation to classification

**Figure 4:**
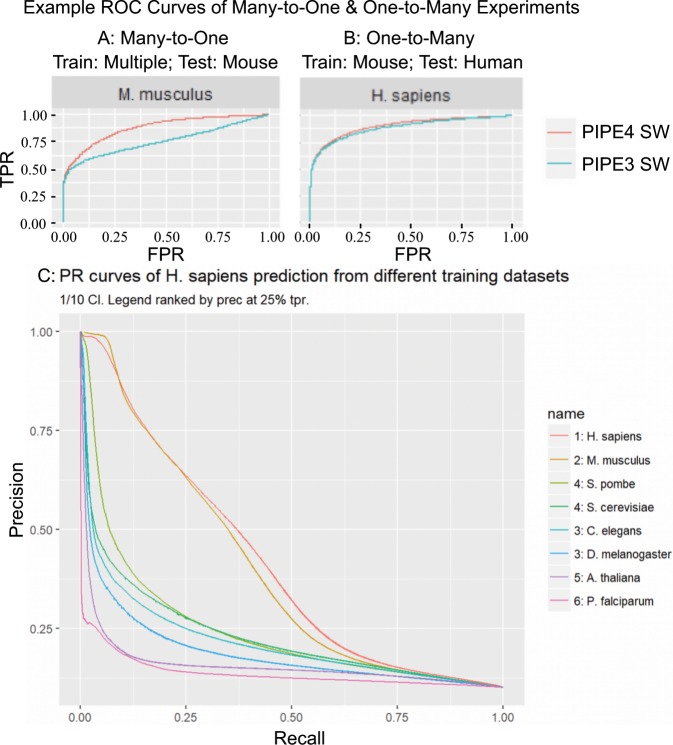
Example ROC and PR Curves of Many-to-One and One-to-Many Experiments. (**A**) depicts the ROC performance when using several organisms to evaluate Mouse PPIs. (**B**) depicts ROC performance using evolutionarily proximal Mouse to predict Human PPIs. (**C**) ranks PR performance when predicting Human PPIs when training on another organism.

One-to-Many cross-species predictions for all 56 pairwise combinations were summarized using AUPRC and Pr@25Re for both PIPE3 and PIPE4. A paired *t*-test on the differences in means revealed a statistically significant difference (*p* < 0.001) with a true increase in mean precision of 1.1% and 1.9% of AUPRC and Pr@25Re respectively for PIPE 4 (Supplementary Table S5). Thus, the new PIPE4 scoring method significantly outperforms the PIPE3 version when using a single training species. Similarly, Many-to-One cross-species predictions for the 8 group-wise combinations were summarized (Supplementary Table S6). A paired *t*-test revealed a statistically significant difference (*p* < 0.01) with a true difference in mean precision estimated to be 9.6% for AUPRC and 16.4% for Pr@25Re. Since the modified SW-score enables the combination of multiple training species, we examined the relationship of evolutionary distance to classification performance. In the majority of cases, evolutionary distance is significantly correlated to performance at the *p* < 0.05 level (16/32, Supplementary Table S7; 19/32, Supplementary Table S8). Qualitatively, the closer related organisms perform well in cross-species predictions with a steep drop-off in performance thereafter (Fig. 4). These findings suggest that One-to-Many cross-species prediction are suitable in both close and distantly-related species whereas Many-to-One predictions are beneficial only in very closely related organisms. While future research in the pursuit of systemically applicable rules are warranted, researchers interested in performing their own inter- and cross-species predictions might consider three key findings from this work when preparing their training datasets:

1. Organisms more closely related to the test species provide higher cross-species prediction accuracy.
2. One-to-Many cross-species prediction are suitable in both close and distantly-related species.
3. Many-to-One cross-species predictions are beneficial only in very closely related organisms.

### Reciprocal perspective for inter- and cross-species predictions

**Figure 5:**
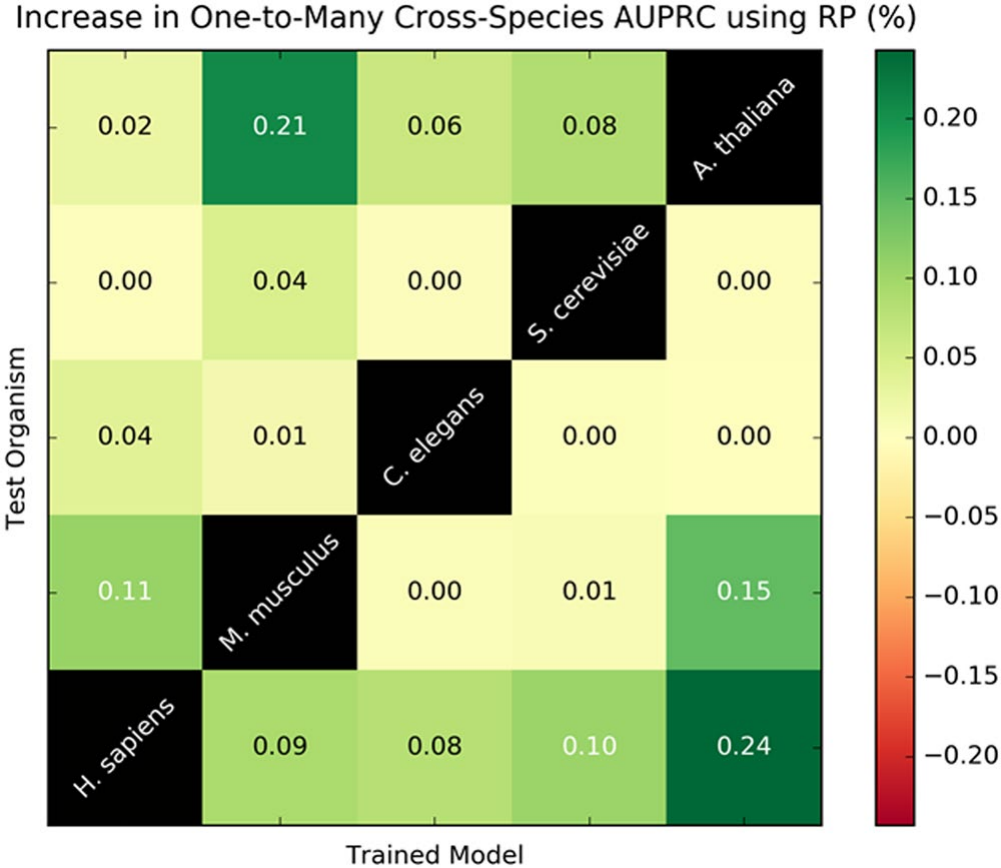
Reciprocal Perspective Increase in AUPRC using One-to-Many Cross-Species Predictions on PIPE4.

When RP is applied to cross-species predictions, only the Rank- and Fold-type features are considered; the original score is excluded from the model. Therefore, we cannot expect a comparable increase in performance as for intra-species application. A non-negative, at times modest increase in AUPRC over PIPE4 is observed for most train/test species pairings (Fig. 5). The largest gains in performance occur for instances where PIPE4 performed particularly poorly (*e.g*. train on *A. thaliana* and test on *H. sapiens*) and where the two organisms have similarly sized proteomes (*e.g. A. thaliana* and *M. musculus*) indicative that these organisms may share similar distributions of protein interaction profiles. By the definitions of the RP method, we would not expect performance gains to correlate with evolutionary distance, but rather with proteome size and the rank-order distribution of relative PPI scores for each protein. Those organisms with the smallest proteome sizes do not lead to sizeable gains in classification performance for organisms with larger proteomes.

The RP meta-method is thus best suited to augmenting inter-species predictive performance and can generally be applied to cross-species schemas with varying degrees of success. Most notably, for cross-species predictions where the initial classifier performs poorly, RP can substantially improve performance and where the initial classifier performs well, modest increase or equivalent performance is expected from applying RP.

### Comparison to the state of the art

**Figure 6:**
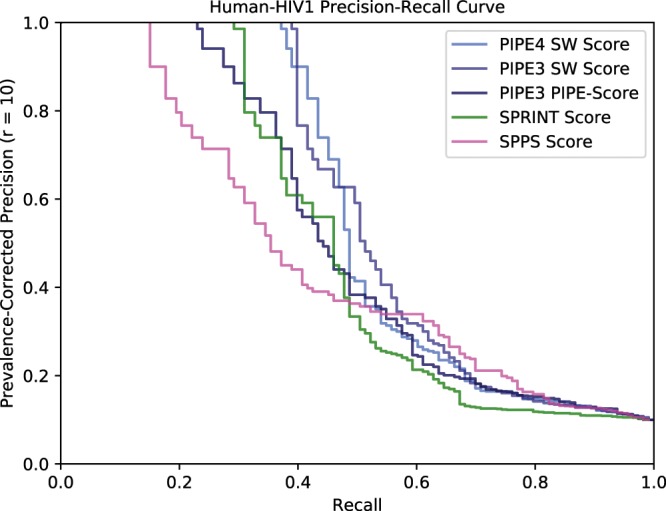
Comparison of PR Curves between PIPE3, PIPE4, SPRINT, and SPPS with on the H. sapiens-HIV1 Inter-Species Interactions.

Comparing the PIPE3, PIPE4, SPRINT, and SPPS methods over the Human-HIV1 inter-species PPI prediction task, we note that the SW-score improves precision over the PIPE3 PIPE-Score and SPRINT score across the range of recall values (Fig. 6). Moreover, the PIPE4 SW-score performance dominates throughout the range of precision values we desire most, *i.e*. Pr ≥ 0.5, where at least half of the positive predictions are true. The PIPE3 and SPRINT scores perform similarly to each other within this range. The SPPS method performs the worst of all methods. The hardware requirements to generate these comprehensive interactomes are comparable between PIPE4 and SPRINT. Evaluating the requisite RAM for the largest prediction task (*G. max*-*H. glycines*) resulted in both methods using ~150GB: 153 and 151, respectively. The SPPS method was not amenable to this study as it far exceeded the limitations on RAM; > 250GB.

The PIPE4 algorithm was explicitly designed to accommodate inter- and cross-species prediction schemas. The vast majority of existing sequence-based PPI predictors were not implemented with inter- and cross-species prediction in mind, including the PIPE3 algorithm. Consequently, the comparison in an inter-species context may be unfair to competing methods. However, in agreement with the findings of Park using PIPE2^10^, we observe that the PIPE3 SW-score consistently outperforms the competing methods, without accounting for the inter-species context (Fig. 6). The SPRINT and SPPS methods may both benefit from adapting their algorithms for use in these complex prediction schemas.

### Summary

With increasingly accurate and efficient PPI predictors applicable to complex prediction schemas, researchers can generate comprehensive interactomes which were originally prohibitive. For example, generating the *H. glycines-G. max* interactome can offer unprecedented insight into the molecular mechanisms between these species (such as with host-pathogen interactions) which may have far-reaching agricultural and economic impact.

In this work we introduced the PIPE4 algorithm, finding it to be significantly faster than its predecessor PIPE3, with speedups of 53.2, 12.5, and 8.4 times observed in *H. sapiens, A. thaliana*, and *S. cerevisiae* respectively. The modified SW-score was shown to improve performance in complex PPI prediction schemas involving cross- and inter-species predictions. The meta-method RP was shown to be effective for these complex cross-species prediction schemas. Finally, competing PPI predictors are expected to exhibit an improvement in predictive performance within these complex schemas if their PPI scoring algorithms can account for the origin of training samples. We published the all-to-all predictions of the inter-species Human-HIV1 predictions for the benefit of the scientific community; available at 10.5683/SP2/PVOTRN.

## Supplementary information

Supplementary Information.

## Data Availability

All datasets are publicly available from the databases (BioGRID, SoyBase and SCNbase) listed in the manuscript. The Human-HIV1 all-to-all predictions are publicly available at 10.5683/SP2/PVOTRN.
