# Supplementary material for: PIPE4: Fast PPI Predictor for Comprehensive Inter- and Cross-Species Interactomes
Source: Sci Rep. 2020 Jan 29;10:1390. doi: 10.1038/s41598-019-56895-w (PMC6989690; doi:10.1038/s41598-019-56895-w)
Supplement: Supplementary file 1 — Supplementary Information. [file 41598_2019_56895_MOESM1_ESM.pdf]

Supplementary File

PIPE4: Fast PPI Predictor for Comprehensive Inter- and Cross-Species Interactomes

Authors: Kevin Dick, Bahram Samanfar, Bradley Barnes, Elroy Cober, Benjamin Mimee, Le Hoa Tan, Stephen J. Molnar, Kyle Biggar, Ashkan Golshani, Frank Dehne, James R. Green

Herein, supporting descriptions and experiments are presented approximately adhering to the order of presentation in the main text. First, a discussion of possible inter-species prediction variants is presented followed by a visual representation of the mathematical notation used to describe the improvements made to the PIPE algorithm. Secondly, the computational improvements of the PIPE4 algorithm are discussed with respect to its predecessor, PIPE3. Thirdly, the cross-species experiments demonstrating improved classification performance of the new Similarity Weighted score are explored with consideration of the origin and influence of training samples on performance outcomes. Thereafter, the inter-species experiments are described in greater detail. Finally, the Reciprocal Perspective methodology is validated with respect to inter- and cross-species prediction schemas.

Inter-Species Prediction Schema Variants

When assembling the training data for inter-species prediction schemas, a number of potential variants exists. Supplementary Fig. 1 illustrates the three most likely training set compositions. Namely, leveraging the *between* species PPIs only (A), leveraging both the *within* species PPIs only (B), or leveraging the *between* and both *within* species PPIs (C). Given the focus of this work on the validation of inter- and cross-species prediction, the schemas depicted in (A) was used. Certainly, the combined schema in (C) with increased magnitude of training samples promises to yield even higher predictive performance, assuming that the training data quality is consistent.

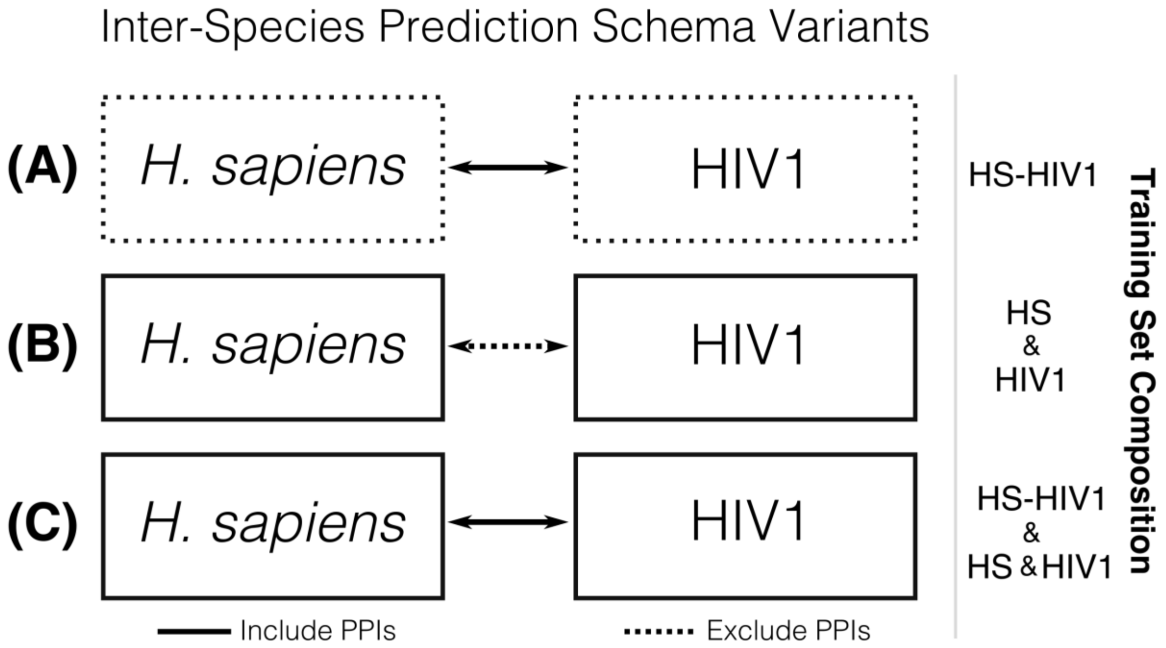

Supplementary Fig. 1. Example of Three Possible Inter-Species Prediction Schema Training Dataset Compositions. (A) leverages only the set of PPIs which have been previously validated occurring *between* the two organisms. (B) leverages the two intra-species sets of PPIs previously validated *within* each of the two organisms. (C) combines the PPI sets from both (A) and (B) producing the largest available PPI training set for two organisms. In this work we use inter-species prediction schema (A) given our focus on validating our methods in a purely inter-species context (*i.e.* not reliant upon available intra-species PPIs).

Benchmarking the New PIPE Algorithm

Timing experiments are performed using the intra-species predictions for three model organisms: *H. sapiens*, *A. thaliana*, and *S. cerevisiae*. These were selected as representing three conventional applications of PIPE in decreasing order of proteome size. Intra-species predictions were performed to ensure use-case compatibility between the PIPE3 and PIPE4 methods. Timing experiments average the time for computing a single PPI over the entire interactome. The relevant benchmark test measure for the intra-species comparison of PIPE3 and PIPE4 are listed in Supplementary Table S1, where the symbol notation are defined in the Methods section of the manuscript. Similarly, the relevant benchmark measures for the combined inter- and cross-species prediction schema are given in Supplementary Table S2. The predictions between *H. glycines* and *G. max* using the PIPE3 would originally have required ~42 days to compute, whereas it was generated in ~2 days using PIPE4, producing a 21.1x speedup. Such speedups are necessary if we were to use PIPE iteratively for purposes such as protein engineering as done in InSIPS. All benchmark measures are tabulated in Supplementary Table S3.

Supplementary Table S1. Intra-Species Benchmark Test Measures

**Benchmark Measure**	*H. sapiens*	*A. thaliana*	*S. cerevisiae*
$\bar{\varphi}$	35.1	21.7	8.2
$\bar{k}$	558	442	450
$\bar{\gamma}$	6.45	3.33	8.15
n	20,236	17,226	6,721
Total known PPIs	66,084	29,035	27,905
Number of positives tested	66,084	29,035	27,905
Number of negatives tested	66,084	3,000,000	3,000,000
Size of comprehensive interactome	204,757,966	148,376,151	22,589,281

Supplementary Table S2. Combined Inter- and Cross-Species Benchmark Test Measures

**Benchmark Measure**	*H. glycines*	*G. max*
$\bar{\varphi}$	104.1	77.8
$\bar{k}$	406	336
$\bar{\gamma}$	0.287	
n	21,868	75,781
Total known PPIs (proxy)	*C. elegans:* 5,476	*A. thaliana*: 29,035
Size of comprehensive interactome	1,657,178,908	

Supplementary Table S3. Inter- and Cross-Species Benchmark Test Measures

**Benchmark Measure**	*H.glycines - G.max*	
	**PIPE4**	**PIPE3**
Database Size (GB)	66	11*
Database Processing (h)	44.01	44.00
Time/pair (s)	0.0037	0.0785
All-to-All Prediction (h)	47.6	1003.8
**Speedup (~x)**	**21.1x**	

* Estimated using Linear Regression assuming database size is proportional to interactome size

Cross-Species Validation Experiments

Previous versions of PIPE have shown success in predicting intra-species PPI in relatively well-studied species with large numbers of experimentally verified PPIs. Cross-species PPI prediction permits experimental data taken from well-studied species to be used to investigate the putative PPI networks of under-studied species which are of research interest yet have very little data available. Through the following experiments, the best practices for cross-species PPI prediction will be examined, including from which species to take training data, for which species valid predictions can then be made, whether using combinations of training species could be advantageous, and validation experiments to demonstrate why the PIPE score needs to be modified for cross-species prediction.

As described in the main text, the cross-species version of PIPE, PIPE4, was created to keep track of the species of origin for each protein and normalizes the landscape score by considering only those PPIs reflected in the training data. To exemplify the importance of this normalization factor, consider the following toy example:

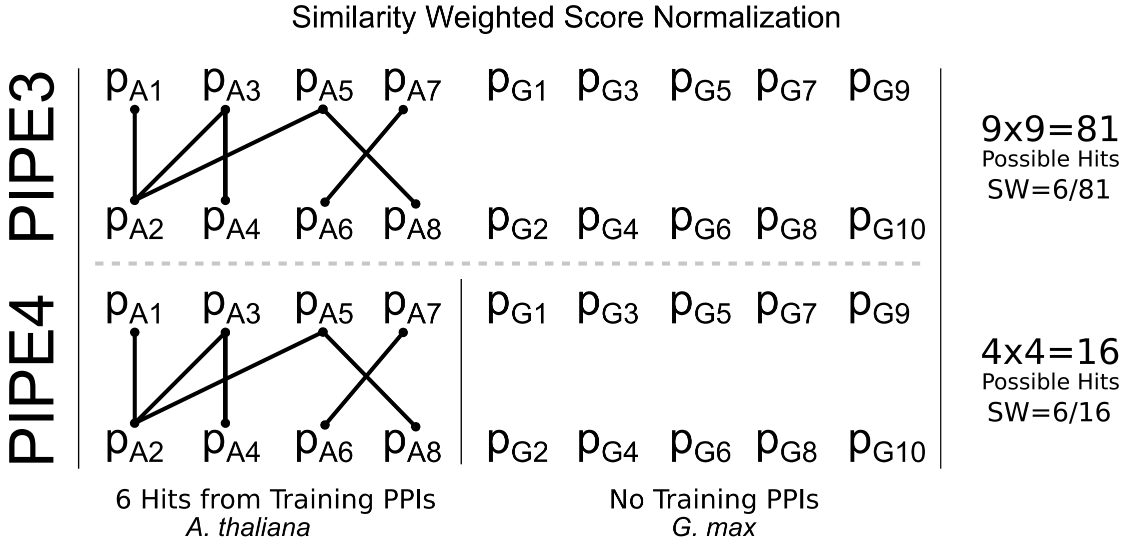

Supplementary Fig. 2. Toy Example Contrasting the PIPE3 and PIPE4 Similarity Weighted Normalization. Each line between the proteins indicates a single "hit" where a window in one protein is similar to a window within the corresponding protein. Hits only exist within the *A. thaliana* proteome as it is a well-studied organisms containing several training PPIs. In PIPE4, this hit count is normalized only by protein pairs within *A. thaliana*.

When using one organism as a proxy for another, the number of possible interactions varies. The original PIPE3 SW score would naively pool the proteomes; however, this does not appropriately represent the true frequency of a window within a proteome. As depicted in Supplementary Fig. 3, the PIPE3 SW would normalize over both proteomes. The prevalence of a given window can vary dramatically between organisms and so the PIPE4 SW score corrects for this by normalizing only over those proteomes which actually have training data; *A. thaliana* only in this case. This normalization becomes more pronounced as the number of species increases and does not strictly scale uniformly across all predicted interactions since each protein window has a varying number of similar proteins. A diverse set of model organisms were considered to examine this normalization factor change further.

The training PPIs for 17 organisms were assembled for the inter- and cross-species experiments (Supplementary Table S4). To control for the amount of available training data, we considered only those organisms with at least 2,000 known PPIs. An equivalently sized set of 2,000 PPIs were randomly subsampled from the set of all known PPIs for each of these resulting eight organisms.

The results from these experiments are summarized using both ROC and PR curves, and the area under the PRC (AU-PRC) and precision at 25% TPR (Pr@25Re) were used as scoring metrics to compare the classification performance of the original PIPE3 SW score and the modified PIPE4 SW score.

Supplementary Table S4. Model Organisms for Inter- and Cross-Species Predictions.

Scientific Name	Common Name	Interactions	Sequences
Homo sapiens	Human	66,084	20,236
Arabidopsis thaliana	Thale cress	29,035	17,226
Saccharomyces cerevisiae	Baker's yeast	27,905	6,721
Drosophila melanogaster	Fruit fly	25,013	8,529
Caenorhabditis elegans	Nematode	5,476	5,891
Schizosaccharomyces pombe	Fission yeast	3,549	5,141
Mus musculus	House mouse	3,402	17,096
Plasmodium falciparum	Malaria-causing parasite	2,250	1,270
Rattus norvegicus	Common rat	531	8,129
Xenopus laevis	African clawed frog	117	169
Solanum lycopersicum	Tomato	98	474
Danio rerio	Zebrafish	94	3,080
Oryza sativa	Asian rice	29	3,832
Gallus gallus	Chicken	28	2,305
Bos taurus	Cow	26	6,026
Glycine max	Soybean	24	429
Candida albicans	Pathogenic yeast	19	1,014

One-to-Many Predictions

The One-to-Many prediction experiments used the training data from one organism to predict interactions for multiple others. This test examines if the modified SW score normalization affected the performance for cross-species predictions when using a single training species. Here, each of the eight species was used to predict cross-species interactions for the remaining seven species. Averaged results over each of the test species are summarized in Supplementary Table S5 and a set of example ROC curves for test species *M. musculus* are depicted in Supplementary Fig. S4. A Student's paired *t*-test under the null hypothesis of equal means yielded a difference in means of 0.011 and 0.019 for AUPRC and Pr@15Re respectively, both with $p < 0.001$.

Supplementary Table S5. Averaged One-to-Many Cross-Species Prediction of the Target Species.

Test Species	AUPRC		Pr@25Re	
	PIPE4	PIPE3	PIPE4	PIPE3
H. sapiens	0.239	0.221	0.284	0.247
A. thaliana	0.163	0.148	0.164	0.142
S. cerevisiae	0.200	0.210	0.225	0.237
S. pombe	0.244	0.248	0.288	0.292
D. melanogaster	0.157	0.142	0.166	0.141
C. elegans	0.190	0.193	0.206	0.209
M. musculus	0.337	0.295	0.422	0.357
P. falciparum	0.127	0.110	0.130	0.111
**Average**	**0.207**	**0.196**	**0.236**	**0.217**

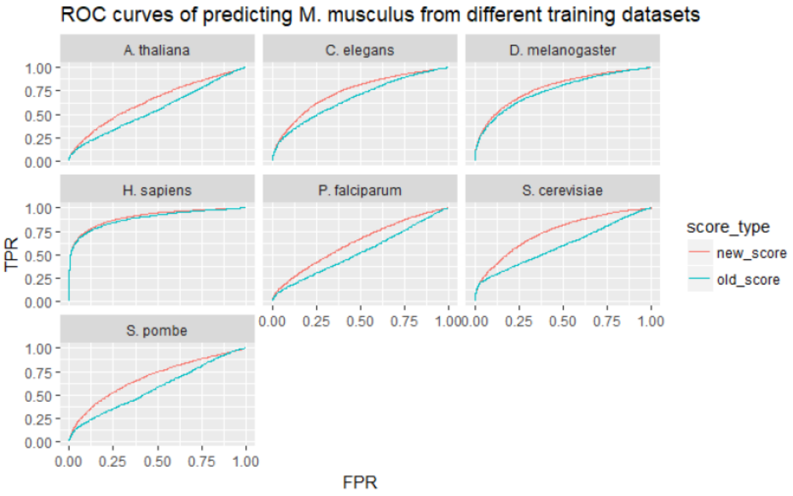

Supplementary Fig. S3. Example ROC Curves of Target Species *M. Musculus* from One-to-Many using Different Training Organisms.

Many-to-One Predictions

The Many-to-One prediction experiments considered pooling the training data for multiple species to then make predictions for another. This test sought to determine whether the normalization change becomes increasingly pronounced with an increase in training species. Here, the interactions for each test species were made with a model trained using PPIs pooled from the other seven species. To correctly reflect this utility in actual application, all available interactions were used here (no sub-sampling was performed for each of the eight species). Results over each of the test species are summarized in Supplementary Table S6 and the ROC curve for each test species is depicted in Supplementary Fig. S5. A Student's paired t-test under the null hypothesis of equal means yielded a difference in means of 0.096 and 0.164 for AUPRC and Pr@15Re respectively, both with $p < 0.05$.

Supplementary Table S6. Many-to-One Cross-Species Performance Metrics.

**Test Species**	**AUPRC**		**Pr@25Re**	
	**PIPE4**	**PIPE3**	**PIPE4**	**PIPE3**
H. sapiens	0.367	0.186	0.494	0.134
A. thaliana	0.214	0.138	0.211	0.104
S. cerevisiae	0.299	0.125	0.370	0.083
S. pombe	0.416	0.298	0.633	0.330
D. melanogaster	0.200	0.156	0.213	0.127
C. elegans	0.249	0.197	0.269	0.160
M. musculus	0.633	0.557	0.959	0.963
P. falciparum	0.140	0.090	0.144	0.078
**Average**	**0.315**	**0.218**	**0.412**	**0.247**

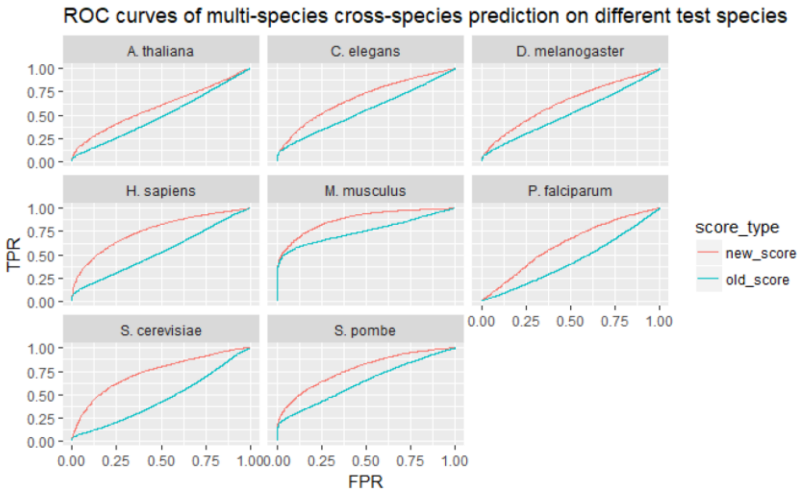

Supplementary Fig. S4. ROC Curves of each Target Species Many-to-One Predictions using the Pooled Training Organisms.

Supplementary Table S7. Statistical tests for rank correlation between the evolutionary rank and the AUPRC rank. 10:1 class imbalance for each test- species and following N=100,000 permutation tests.

	**Kendall's Tau-b Correlation**			**Spearman's Rank Correlation**		
**Test Species**	Corr. Coeff.	*p*-value from R	*p*-value perm. tests	Corr. Coeff.	*p*-value from R	*p*-value perm. tests
H. sapiens	0.67	**0.024**	**0.014**	0.80	**0.018**	**0.012**
A. thaliana	0.68	**0.033**	**0.018**	0.76	**0.027**	**0.017**
S. cerevisiae	0.32	0.288	0.184	0.34	0.406	0.206
S. pombe	0.48	0.111	0.076	0.58	0.129	0.069
D. melanogaster	0.82	**0.006**	**0.002**	0.93	**0.001**	**0.001**
C. elegans	0.44	0.132	0.087	0.52	0.188	0.099
M. musculus	0.82	**0.006**	**0.002**	0.89	**0.003**	**0.003**
P. falciparum	0.5	0.127	0.124	0.58	0.134	0.125

Supplementary Table S8. Statistical tests for rank correlation between the evolutionary rank and the Pr@25Re rank. 10:1 class imbalance for each test- species and following N=100,000 permutation tests.

	**Kendall's Tau-b Correlation**			**Spearman's Rank Correlation**		
**Test Species**	Corr. Coeff.	p-value from R	p-value perm. tests	Corr. Coeff.	p-value from R	p-value perm. tests
H. sapiens	0.67	**0.024**	**0.015**	0.8	**0.018**	**0.012**
A. thaliana	0.68	**0.033**	**0.018**	0.76	**0.027**	**0.018**
S. cerevisiae	0.4	0.184	0.121	0.41	0.318	0.165
S. pombe	0.56	0.063	**0.041**	0.65	0.083	**0.042**
D. melanogaster	0.74	**0.012**	**0.006**	0.88	**0.004**	**0.003**
C. elegans	0.52	0.079	**0.049**	0.58	0.133	0.07
M. musculus	0.82	**0.006**	**0.002**	0.89	**0.003**	**0.002**
P. falciparum	0.5	0.127	0.125	0.58	0.134	0.125

Reciprocal Perspective Inter- and Cross-Species Validation Experiments

The Reciprocal Perspective (RP) meta-predictor was originally developed for intra-species prediction schemas; however, RP is defined in such a way that it can be applied to inter-species, cross-species, and combined schemas. This work, in addition to presenting the updated version of the PIPE algorithm, sought to validate the RP meta-predictor for these contexts and exemplify the achievable improvements in classification performance. Thus, not only do we present an ultra-fast variant of the PIPE algorithm which is broadly applicable to intra-, inter-, and cross-species prediction schemas, we also demonstrate how large improvements in predictive performance can be achieved using the RP meta-predictor.

Expanding upon the PIPE4 cross-species validation experiments, we generated RP classifiers trained on one organism and evaluated their performance on others. The RP method extracts a number of context-based features from the comprehensive set of all predicted PPIs and applies a cascaded machine learning layer to further differentiate the positive and negative class. The vanilla version of the RP method can easily be applied to the intra- and inter-species prediction schema however an interesting facet of the PIPE SW score is that its range of values can vary dramatically between organisms, largely due to the number of training PPIs available and the prevalence of windows within each organism's proteomes. As such, certain organisms have inflated scores with respect to others. To control for these score-based biases, we restricted our analysis to RP features which are based solely upon rank (*i.e.* Rank-Type) and fold-difference from the protein-specific baseline (*i.e.* Fold-Type) and did not leverage the score-based features (*i.e.* Score-Type). We have previously shown that the joint combination of these features can lead to statistically significant improvement in predictive performance. Given the limited set of features here, we can only expect modest increases in predictive performance.

Cross-Species Validation Experiments

The RP features for the cross-species validations experiments were reduced to only the Rank- and Fold-Type. Here, a subset of five model organisms were considered for One-to-Many predictions. This experiment sought to examine the expected improvement in predictive performance over the PIPE4 cross-species experiments. The baseline performance was therefore established as the average AUPRC from the PIPE4 cross-species experiments. The reduced set of RP features were extracted and a Random Forest classifier trained. The model was then evaluated on each of the four other organisms. The AUPRC was noted and this process was repeated for 1,000 bootstrap iterations to produce a distribution of AUPRCs and the average was reported. The difference in average AUPRC was depicted in Fig. 3 in the main text.
